# Suicide Rates by Industry and Occupation — National Vital Statistics System, United States, 2021

**DOI:** 10.15585/mmwr.mm7250a2

**Published:** 2023-12-15

**Authors:** Aaron Sussell, Cora Peterson, Jia Li, Arialdi Miniño, Kenneth A. Scott, Deborah M. Stone

**Affiliations:** ^1^Spokane Mining Research Division, National Institute for Occupational Safety and Health, CDC; ^2^Division of Injury Prevention, National Center for Injury Prevention and Control, CDC; ^3^Division of Field Studies and Engineering, National Institute for Occupational Safety and Health, CDC; ^4^Division of Vital Statistics, National Center for Health Statistics, CDC; ^5^Western States Division, National Institute for Occupational Safety and Health, CDC.

**Summary**

**What is already known about this topic?**

The 2021 suicide rate among U.S. persons of working age is approximately 33% higher than it was 2 decades ago.

**What is added by this report?**

Data from 49 states were used to calculate suicide rates by sex for all major and detailed industry and occupational groups. Major industry groups with the highest suicide rates included Mining; Construction; Other Services; Arts, Entertainment, and Recreation; and Agriculture, Forestry, Fishing, and Hunting. Major occupation groups with higher suicide rates included Construction and Extraction; Farming, Fishing, and Forestry; Personal Care and Service; Installation, Maintenance, and Repair; and Arts, Design, Entertainment, Sports, and Media.

**What are the implications for public health practice?**

Variations in suicide rates indicate risk disparities by workers’ industry and occupation. Workplaces can be important settings for suicide prevention. CDC provides evidence-based suicide prevention strategies in its Suicide Prevention Resource for Action and Critical Steps Your Workplace Can Take Today to Prevent Suicide, National Institute for Occupational Safety and Health Science Blog.

## Abstract

The suicide rate among the U.S. working-age population has increased approximately 33% during the last 2 decades. To guide suicide prevention strategies, CDC analyzed suicide deaths by industry and occupation in 49 states, using data from the 2021 National Vital Statistics System. Industry (the business activity of a person’s employer or, if self-employed, their own business) and occupation (a person’s job or the type of work they do) are distinct ways to categorize employment. The overall suicide rates by sex in the civilian noninstitutionalized working population were 32.0 per 100,000 among males and 8.0 per 100,000 among females. Major industry groups with the highest suicide rates included Mining (males = 72.0); Construction (males = 56.0; females = 10.4); Other Services (e.g., automotive repair; males = 50.6; females = 10.4); Arts, Entertainment, and Recreation (males = 47.9; females = 15.0); and Agriculture, Forestry, Fishing, and Hunting (males = 47.9). Major occupation groups with the highest suicide rates included Construction and Extraction (males = 65.6; females = 25.3); Farming, Fishing, and Forestry (e.g., agricultural workers; males = 49.9); Personal Care and Service (males = 47.1; females = 15.9); Installation, Maintenance, and Repair (males = 46.0; females = 26.6); and Arts, Design, Entertainment, Sports, and Media (males = 44.5; females = 14.1). By integrating recommended programs, practices, and training into existing policies, workplaces can be important settings for suicide prevention. CDC provides evidence-based suicide prevention strategies in its Suicide Prevention Resource for Action and Critical Steps Your Workplace Can Take Today to Prevent Suicide, NIOSH Science Blog.

## Introduction

In 2021, a total of 37,602 persons (17.8 per 100,000 population) of working age (16–64 years, employed or unemployed) in the United States died by suicide, a rate increase of approximately 33% since 2001 (13.4).* To guide suicide prevention strategies among the working-age population, CDC analyzed 2021 suicide deaths by industry and occupation.

## Methods

### Data Source 

The 2021 National Vital Statistics System (NVSS) restricted-use mortality file includes 37,435 suicide decedents aged 16–64 years who resided and died in one of 49 states (death certificate reporting from Rhode Island and the District of Columbia did not include usual industry and occupation) (*1*). This report includes 30,015 decedents (80.2% of total) whose usual industry or occupation reported on the death certificate was not military, nonpaid (i.e., homemaker, volunteer, student, retired, did not work, child, disabled, patient, or inmate), or unclassifiable (7,420). Industry (the business activity of a person’s employer or, if self-employed, their own business) and occupation (a person’s job or the type of work they do) are distinct ways to categorize employment (*2*). Major industry and occupation classifications encompass all jobs in the U.S. economy; detailed industry and occupation groups are subcategories of major groups that define job types with more specificity.

### Data Analyses 

Population estimates for rate denominators were states’ civilian, noninstitutionalized, currently employed, working population counts for persons aged 16–64 years from the 2021 American Community Survey Public Use Microdata Sample. Replicate weight standard errors for those counts were used to calculate 95% CIs. Rates were calculated by sex for major and detailed industry and occupational groups with ≥20 decedents and compared with the total sex-specific civilian noninstitutionalized working population. Industry and occupational groups with suicide rates significantly (*α* = 0.05) higher by sex than the respective total civilian noninstitutionalized working population (all industries or all occupations combined) were identified as having elevated rates when the lower end of the group’s 95% CI exceeded the study population rate point estimate. This analysis used the population rate point estimates for males and females as constant comparative values, in keeping with a previously published report, so that all comparisons used common points of reference (*3*). Rates were not calculated for “not specified” and “other general” industry groups because of inadequate alignment with available data on working population size. U.S. Census Bureau 2012 industry and occupation codes as reported in 2021 NVSS were matched to U.S. Census Bureau 2017 industry and 2018 occupation codes as reported in the 2021 American Community Survey using standard definitions (*4*). Statistical analyses were conducted using SAS software (version 9.4; SAS Institute). This activity was reviewed by CDC, deemed research not involving human subjects, and was conducted consistent with applicable federal law and CDC policy.^†^

## Results

### Suicide Rates by Major Industry and Occupation Groups

The overall 2021 suicide rate per 100,000 in the U.S. civilian noninstitutionalized working population was 32.0 among males and 8.0 among females. Compared with the entire civilian noninstitutionalized working population, suicide rates for males, females, or both were elevated in nine of 20 major industry groups: Mining (males = 72.0); Construction (males = 56.0; females = 10.4); Other Services (e.g., automotive repair; males = 50.6; females = 10.4); Arts, Entertainment, and Recreation (males = 47.9; females = 15.0), Agriculture, Forestry, Fishing, and Hunting (males = 47.9); Transportation and Warehousing (males = 35.5); Administrative and Support and Waste Management and Remediation Services (males = 35.2); Accommodation and Food Services (males = 34.7; females = 11.1); and Health Care and Social Assistance (females = 8.5) (Table 1). Suicide rates by sex were elevated in 12 of 22 major occupational groups: Construction and Extraction (males = 65.6; females = 25.3); Farming, Fishing, and Forestry (e.g., agricultural workers; males = 49.9); Personal Care and Service (males = 47.1; females = 15.9); Installation, Maintenance, and Repair (males = 46.0; females = 26.6); Arts, Design, Entertainment, Sports, and Media (males = 44.5; females = 14.1); Building and Grounds Cleaning and Maintenance (males = 37.6); Production (males = 36.0); Transportation and Material Moving (males = 34.9); Protective Service (males = 34.8; females = 11.6); Food Preparation and Serving Related (females = 10.6); Healthcare Practitioners and Technical (females = 9.4); and Sales and Related (females = 8.9) (Table 2).

**TABLE 1:** Suicide rates,* by sex, for 20 major industry groups and detailed groups with rates higher than all industries combined — National Vital Statistics System, United States,^†^ 2021

Industry group^§^	Rate (95% CI)	
	Males	Females
**All industries**	**32.0 (31.6–32.4)**	**8.0 (7.8–8.2)**
Accommodation and food services	34.7 (32.9–36.4)^¶^	11.1 (10.2–12.1)^¶^
Drinking places and alcoholic beverages	56.5 (35.7–85.8)^¶^	28.7 (16.7–46.6)^¶^
Restaurants and other food services	34.6 (32.7–36.5)^¶^	11.3 (10.3–12.3)^¶^
Administrative and support and waste management and remediation services	35.2 (33.2–37.2)^¶^	6.8 (5.7–7.8)
Investigation and security services	44.7 (38.9–50.5)^¶^	9.4 (5.3–15.8)
Landscaping services	52.3 (47.8–56.7)^¶^	—**
Agriculture, forestry, fishing, and hunting	47.9 (44.0–51.8)^¶^	10.9 (7.5–15.4)
Crop production	46.2 (41.0–51.4)^¶^	9.2 (5.3–15.0)
Fishing, hunting, and trapping	111.7 (68.3–174.3)^¶^	—
Logging	113.8 (77.8–161.7)^¶^	—
Arts, entertainment, and recreation	47.9 (44.3–51.6)^¶^	15.0 (12.8–17.1)^¶^
Performing arts, spectator sports, and related industries^††^	126.2 (113.3–139.2)^¶^	46.5 (38.0–55.0)^¶^
Construction	56.0 (54.4–57.6)^¶^	10.4 (8.5–12.3)^¶^
Education services	11.6 (10.6–12.7)	4.0 (3.6–4.4)
Finance and insurance	16.6 (15.2–18.0)	5.7 (4.9–6.4)
Health care and social assistance	20.9 (19.5–22.2)	8.5 (8.1–9.0)^¶^
Home health care services	32.4 (23.0–44.5)	10.3 (8.3–12.2)^¶^
Hospitals^††^	22.1 (19.9–24.4)	11.8 (10.9–12.8)^¶^
Nursing care facilities (skilled nursing facilities)	27.4 (19.9–36.9)	12.6 (10.5–14.7)^¶^
Information	22.5 (20.2–24.8)	8.0 (6.1–10.2)
Management of companies and enterprises	—	—
Manufacturing	29.8 (28.7–30.9)	6.5 (5.8–7.3)
Aerospace products and parts manufacturing	147.5 (116.8–178.2)^¶^	—
Ship and boat building	46.0 (32.4–63.6)^¶^	—
Mining	72.0 (64.0–80.0)^¶^	—
Coal mining	83.9 (50.2–133.1)^¶^	—
Oil and gas extraction and support activities for mining^§^	73.9 (63.8–84.0)^¶^	—
Other services (except public administration)	50.6 (48.0–53.2)^¶^	10.4 (9.3–11.5)^¶^
Automotive repair and maintenance	77.7 (72.1–83.2)^¶^	—
Barber shops	56.7 (36.2–85.5)^¶^	—
Beauty salons	38.8 (22.4–63.4)	17.3 (14.4–20.3)^¶^
Car washes	49.6 (34.7–69.0)^¶^	—
Commercial and industrial machinery and equipment repair and maintenance	81.7 (70.0–93.3)^¶^	—
Nail salons and other personal care services	55.2 (38.2–77.6)^¶^	14.7 (10.4–20.4)^¶^
Personal and household goods repair and maintenance	53.8 (37.0–76.1)^¶^	—
Other personal services	32.4 (21.4–47.5)	12.6 (8.3–18.4)^¶^
Private households	69.9 (42.6–109.3)^¶^	8.9 (6.1–12.5)
Professional, scientific, and technical services	20.2 (19.1–21.3)	8.0 (7.3–8.8)
Legal services	23.7 (19.7–27.7)	10.6 (8.2–13.5)^¶^
Specialized design services	40.4 (27.4–57.8)	20.7 (13.6–30.3)^¶^
Veterinary services	42.1 (25.5–66.1)	12.8 (8.9–17.8)^¶^
Public administration	25.8 (24.2–27.5)	6.7 (5.8–7.6)
Real estate and rental and leasing	22.7 (20.1–25.3)	8.2 (6.6–9.8)
Retail trade	23.4 (22.4–24.5)	8.1 (7.5–8.8)
Transportation and warehousing	35.5 (33.9–37.1)^¶^	8.6 (7.3–9.9)
Services incidental to transportation	40.8 (35.5–46.2)^¶^	11.0 (6.3–18.0)
Truck transportation	52.4 (48.8–56.1)^¶^	11.5 (7.1–17.9)
Warehousing and storage	46.5 (41.0–51.9)^¶^	9.9 (6.4–14.7)
Utilities	34.2 (30.5–37.9)	10.8 (6.8–16.4)
Wholesale trade	14.5 (12.9–16.0)	3.8 (2.6–5.4)
Recyclable material merchant wholesalers	56.7 (33.6–90.7)^¶^	—

* Rates are per 100,000 civilian, noninstitutionalized working persons aged 16–64 years from the 2021 American Community Survey.

^†^ Rhode Island and the District of Columbia are not included.

^§^ U.S. Census Bureau 2017 industry titles are from the 2021 American Community Survey unless otherwise noted. U.S. Census Bureau codes 0370 and 0490 were combined because nearly all workers classified as 0490 work in oil and gas extraction, and they are the majority of workers in that industry.

^¶^ Rates are significantly (*α* = 0.05) higher than the total civilian noninstitutionalized working population (all industries or occupations combined) and were identified as having elevated rates when the lower end of the group’s 95% CI exceeded the study population rate point estimate. Caution should be taken when interpreting rate point estimates for detailed groups with wide 95% CIs, which can be due to relatively low numbers of decedents and working populations.

** Rate was not calculated if industry or occupation group codes were irreconcilable between data sources; the group was a composite category with low specificity (e.g., “Not Specified Retail Trade”), number of deaths was fewer than 20, or population estimate was unavailable.

^††^ This is a U.S. Census Bureau 2012 industry title.

**TABLE 2:** Suicide rates,* by sex, for 22 major occupation groups and detailed groups with rates higher than all occupations combined — National Vital Statistics System, United States,^†^ 2021

Occupation group^§^	Rate (95% CI)	
	Males	Females
**All occupations**	**32.0 (31.6–32.4)**	**8.0 (7.8–8.2)**
Architecture and engineering	21.9 (20.1–23.7)	7.5 (5.2–10.6)
Surveyors, cartographers, and photogrammetrists	119.4 (66.7–199.7)^¶^	—**
Arts, design, entertainment, sports, and media	44.5 (41.0–48.0)^¶^	14.1 (12.2–16.0)^¶^
Artists and related workers	93.3 (76.2–110.5)^¶^	45.3 (31.0–64.2)^¶^
Entertainers and performers, sports and related workers, all other^††^	114.5 (61.5–197.2)^¶^	—
Musicians, singers, and related workers^††^	138.7 (113.0–164.4)^¶^	—
Writers and authors	53.1 (34.4–79.1)^¶^	22.8 (13.8–35.9)^¶^
Building and grounds cleaning and maintenance	37.6 (35.3–39.8)^¶^	7.5 (6.3–8.7)
First-line supervisors of landscaping, lawn service, and groundskeeping workers	55.7 (39.3–77.0)^¶^	—
Grounds maintenance workers^††^	46.7 (42.6–50.9)^¶^	—
Business and financial operations	13.5 (12.4–14.7)	4.6 (4.0–5.2)
Community and social services	17.9 (15.0–20.9)	8.1 (6.8–9.5)
Social workers^¶^	29.7 (18.9–44.8)	12.0 (9.0–15.7)^¶^
Computer and mathematical	16.1 (14.9–17.3)	4.9 (3.7–6.4)
Construction and extraction	65.6 (63.7–67.6)^¶^	25.3 (18.2–34.3)^¶^
Brickmasons, blockmasons, and stonemasons	63.1 (45.5–85.6)^¶^	—
Carpenters	69.4 (64.2–74.6)^¶^	—
Carpet, floor, and tile installers and finishers	49.5 (33.5–71.1)^¶^	—
Construction equipment operators	58.6 (50.3–66.9)^¶^	—
Construction laborers	91.0 (86.0–95.9)^¶^	38.6 (22.0–63.7)^¶^
Derrick, rotary drill, and service unit operators, oil and gas	116.8 (60.0–208.0)^¶^	—
Earth drillers, except oil and gas^††^	64.4 (33.1–115.3)^¶^	—
Electricians	52.1 (47.1–57.1)^¶^	—
First-line supervisors of construction trades and extraction workers	61.7 (55.0–68.4)^¶^	—
Glaziers	69.6 (39.3–115.6)^¶^	—
Painters and paperhangers	44.1 (37.8–50.4)^¶^	—
Pipelayers, plumbers, pipefitters, and steamfitters^††^	49.4 (43.5–55.3)^¶^	—
Roofers	79.9 (66.5–93.2)^¶^	—
Structural iron and steel workers	86.1 (57.2–125.3)^¶^	—
Other extraction workers	128.7 (83.4–190.7)^¶^	—
Education, training, and library	11.2 (9.8–12.5)	4.1 (3.6–4.6)
Farming, fishing, and forestry	49.9 (44.2–55.5)^¶^	—
Fishing and hunting workers	130.6 (78.8–205.8)^¶^	—
Logging workers	161.1 (106.8–234.7)^¶^	—
Miscellaneous agricultural workers	38.1 (32.5–43.6)^¶^	—
Food preparation and serving related	32.2 (30.3–34.2)	10.6 (9.6–11.6)^¶^
Bartenders	47.0 (33.5–64.5)^¶^	23.8 (16.3–33.7)^¶^
Chefs and head cooks	66.5 (57.2–75.7)^¶^	32.9 (19.8–52.0)^¶^
Cooks	35.9 (32.4–39.4)^¶^	7.7 (5.6–10.3)
Waiters and waitresses	32.4 (27.3–37.6)	16.2 (13.8–18.5)^¶^
Healthcare practitioners and technical	22.0 (20.0–23.9)	9.4 (8.7–10.2)^¶^
Health practitioner support technologists and technicians^††^	25.0 (17.6–34.5)	10.8 (8.1–14.1)^¶^
Registered nurses	28.4 (22.9–33.9)	11.3 (10.0–12.5)^¶^
Healthcare support	19.7 (16.4–22.9)	7.9 (7.0–8.7)
Massage therapists	—	25.8 (16.2–39.5)^¶^
Nursing, psychiatric, and home health aides^††^	38.2 (28.4–50.4)	11.2 (9.5–12.9)^¶^
Personal care aides	32.8 (24.3–43.6)	10.9 (8.9–12.9)^¶^
Installation, maintenance, and repair	46.0 (44.0–48.1)^¶^	26.6 (18.0–38.2)^¶^
Automotive body and related repairers	68.5 (49.6–92.6)^¶^	—
Automotive service technicians and mechanics	80.6 (74.0–87.2)^¶^	—
Bus and truck mechanics and diesel engine specialists	40.1 (32.8–47.5)^¶^	—
Computer, automated teller, and office machine repairers	65.7 (46.9–90.0)^¶^	—
Heating, air conditioning, and refrigeration mechanics and installers	50.4 (43.3–57.4)^¶^	—
Industrial and refractory machinery mechanics	39.8 (33.1–46.6)^¶^	—
Millwrights	73.2 (45.1–113.4)^¶^	—
Legal	20.5 (17.2–23.8)	8.0 (6.1–10.4)
Life, physical, and social science	21.9 (18.7–25.0)	7.0 (5.1–9.4)
Agricultural and food scientists	173.1 (94.1–294.6)^¶^	—
Management	21.1 (20.2–22.1)	5.8 (5.2–6.3)
Construction managers	41.1 (36.6–45.6)^¶^	—
Farmers, ranchers, and other agricultural managers	52.1 (44.8–59.4)^¶^	—
Food service managers	38.2 (32.3–44.0)^¶^	12.3 (8.7–16.9)^¶^
Office and administrative support	23.2 (21.8–24.7)	6.8 (6.3–7.2)
Postal service clerks	58.2 (32.2–98.5)^¶^	—
Personal care and service	47.1 (42.3–52.0)^¶^	15.9 (14.3–17.4)^¶^
Animal caretakers	32.2 (17.6–55.1)	14.2 (8.9–21.7)^¶^
Barbers	58.8 (38.4–86.9)^¶^	—
Hairdressers, hairstylists, and cosmetologists	37.7 (19.8–66.2)	17.0 (13.8–20.2)^¶^
Personal care and service workers, all other^††^	95.4 (59.2–146.9)^¶^	—
Production	36.0 (34.4–37.6)^¶^	8.0 (6.9–9.2)
Machinists	62.5 (53.4–71.6)^¶^	—
Production workers, all other^††^	35.0 (31.1–38.8)	13.0 (9.0–18.2)^¶^
Welding, soldering, and brazing workers	81.9 (73.8–89.9)^¶^	—
Protective service	34.8 (32.4–37.3)^¶^	11.6 (8.8–14.9)^¶^
Bailiffs, correctional officers, and jailers^††^	41.3 (33.5–49.0)^¶^	—
Security guards and gambling surveillance officers	46.3 (40.7–51.8)^¶^	10.7 (6.2–17.5)
Sales and related	25.7 (24.5–26.9)	8.9 (8.2–9.6)^¶^
Retail salespersons	44.3 (40.6–47.9)^¶^	15.1 (13.0–17.3)^¶^
Transportation and material moving	34.9 (33.6–36.1)^¶^	8.8 (7.6–10.0)
Crane and tower operators	63.4 (39.7–97.1)^¶^	—
Driver/Sales workers and truck drivers	33.2 (31.2–35.3)	15.9 (10.6–23.1)^¶^
Laborers and freight, stock, and material movers, hand	68.7 (64.9–72.5)^¶^	17.9 (14.4–21.4)^¶^

* Rates are per 100,000 civilian, noninstitutionalized working persons aged 16–64 years from the 2021 American Community Survey.

^†^ Rhode Island and the District of Columbia are not included.

^§^ U.S. Census Bureau 2018 occupation titles are from the 2021 American Community Survey unless otherwise noted.

^¶^ Rates are significantly (*α* = 0.05) higher than the total civilian noninstitutionalized working population (all industries or occupations combined) and were identified as having elevated rates when the lower end of the group’s 95% CI exceeded the study population rate point estimate. Caution should be taken when interpreting rate point estimates for detailed groups with wide 95% CIs, which can be due to relatively low numbers of decedents and working populations.

** Rate was not calculated if industry or occupation group codes were irreconcilable between data sources; the group was a composite category with low specificity (e.g., “Not Specified Retail Trade”), number of deaths was fewer than 20, or population estimate was unavailable.

^††^ This is a U.S. Census Bureau 2012 occupation title.

### Suicide Rates by Detailed Industry Groups

Among 254 detailed industry groups, suicide rates were elevated for males, females, or both in 31 groups (Table 1). The five detailed industry groups with the highest suicide rates among males were Aerospace Products and Parts Manufacturing (147.5 per 100,000 population); Performing Arts, Spectator Sports, and Related Industries (126.2); Logging (113.8); Fishing, Hunting, and Trapping (111.7); and Coal Mining (83.9). Among females, the five detailed industry groups with the highest rates were Performing Arts, Spectator Sports, and Related Industries (46.5); Drinking Places, Alcoholic Beverages (28.7); Specialized Design Services (20.7); Beauty Salons (17.3); and Nail Salons and Other Personal Care Services (14.7).

### Suicide Rates by Detailed Occupation Groups

Among 492 detailed occupation groups, suicide rates were elevated for males, females, or both in 60 of those groups (Table 2). The five detailed occupational groups with the highest suicide rates among males were Agricultural and Food Scientists (173.1); Logging Workers (161.1); Musicians, Singers, and Related Workers (138.7); Fishing and Hunting Workers (130.6); and Other Extraction Workers (128.7). Among females, the five detailed occupational groups with the highest rates were Artists and Related Workers (45.3); Construction Laborers (38.6); Chefs and Head Cooks (32.9); Massage Therapists (25.8); and Bartenders (23.8). Rates for all major and detailed groups are available (Supplementary Table 1, https://stacks.cdc.gov/view/cdc/136410) (Supplementary Table 2, https://stacks.cdc.gov/view/cdc/136411).

## Discussion

Similar to an analysis of 32 states using the 2016 National Violent Death Reporting System, the current report identified suicide rates for males, females, or both in multiple major industry groups that were higher than in the total civilian noninstitutionalized working population. These industry groups included Mining; Construction; Other Services (e.g., automotive repair); Arts, Entertainment, and Recreation; Agriculture, Forestry, Fishing, and Hunting. Major occupational groups with elevated rates include Construction and Extraction; Farming, Fishing, and Forestry (e.g., agricultural workers); Personal Care and Service; Installation, Maintenance, and Repair; Arts, Design, Entertainment, Sports, and Media (*3*). Although relative comparisons of suicide rates in this manner can be useful for prevention purposes, these results should not overshadow the larger context that the suicide rate in the U.S. working-age population overall has increased by approximately one third during the last 2 decades, pointing to the need for more research on causal factors and workplace- and community-based prevention. Suicide risk is associated with low-skilled jobs (*5*), lower educational attainment (*6*), lower absolute and relative socioeconomic status (*7*), work-related access to lethal means of suicide (*8*), and job stress, including poor supervisory and colleague support, low job control, and job insecurity (*9*). Starting in 2020, CDC included data on decedents’ usual industry and occupation from death certificates in the publicly available NVSS, providing important opportunities to monitor and address preventable mortality in the U.S. working population.

### Limitations

The findings in this report are subject to at least four limitations. First, this study did not address confounding factors other than sex (e.g., age, race and ethnicity, educational attainment, and other nonwork factors) that might account for different suicide rates among and within industry or occupational groups. Second, this report addressed suicide only among decedents with classifiable paid employment reported on the death certificate; previous research has shown that job reporting on death certificates is associated with decedents’ sociodemographic characteristics (*10*). Third, this analysis did not address suicide among decedents with military employment nor decedents aged ≥65 years. Finally, the numerator and denominator data were not a direct match for rate calculation; death certificates reflect decedents’ usual industry and occupation, whereas available population size data refer to the number of workers by current job.

### Public Health Implications

Variations in suicide rates indicate risk disparities by workers’ industry and occupation. Workplaces can integrate evidence-based suicide prevention strategies and training into existing policies and procedures, such as limiting access to lethal means, providing peer support, increasing access to mental health services, and reducing stigma to encourage easier access to quality care. Suicide is preventable through a comprehensive public health approach that addresses the many factors associated with suicide, including those associated with industry and occupation. CDC provides guidance in its Suicide Prevention Resource for Action (https://www.cdc.gov/suicide/resources/prevention.html) and Critical Steps Your Workplace Can Take Today to Prevent Suicide, NIOSH Science Blog (https://blogs.cdc.gov/niosh-science-blog/2023/03/15/preventing-workplace-suicide). For persons in crisis, help is available through the Substance Abuse and Mental Health Services Administration’s 988 Suicide and Crisis Lifeline (https://www.988lifeline.org or by texting or calling 988).

## Footnotes

*
https://bit.ly/46GuCMA

† 45 C.F.R. part 46; 21 C.F.R. part 56; 42 U.S.C. Sect. 241(d), 5 U.S.C. Sect. 552a, 44 U.S.C. Sect. 3501 et seq.
